# Risk of atopic dermatitis in periodontitis patients with and without dental scaling: A retrospective cohort study

**DOI:** 10.1371/journal.pone.0333877

**Published:** 2025-10-15

**Authors:** Ming-Che Chiang, Chuen-Chau Chang, Chia-Yen Lee, Chun-Chieh Yeh, Yu-Chen Huang, Ta-Liang Chen, Chien-Chang Liao

**Affiliations:** 1 Department of Dermatology, Wan Fang Hospital, Taipei Medical University, Taipei, Taiwan; 2 Department of Dermatology, En Chu Kong Hospital, New Taipei City, Taiwan; 3 Department of Anesthesiology, Taipei Medical University Hospital, Taipei, Taiwan; 4 Anesthesiology and Health Policy Research Center, Taipei Medical University Hospital, Taipei, Taiwan; 5 Department of Anesthesiology, School of Medicine, College of Medicine, Taipei Medical University, Taipei, Taiwan; 6 Department of Dentistry, Taiwan Adventist Hospital, Taipei, Taiwan; 7 Department of Surgery, China Medical University Hospital, Taichung, Taiwan; 8 Department of Surgery, University of Illinois, Chicago, Illinois, United States of America; 9 Department of Dermatology, School of Medicine, College of Medicine, Taipei Medical University, Taipei, Taiwan; 10 Research Center of Big Data and Meta-Analysis, Wan Fang Hospital, Taipei Medical University, Taipei, Taiwan; 11 Department of Anesthesiology, Wan Fang Hospital, Taipei Medical University, Taipei, Taiwan; 12 School of Chinese Medicine, College of Chinese Medicine, China Medical University, Taichung, Taiwan; Danube Private University, AUSTRIA

## Abstract

**Background:**

Both atopic dermatitis (AD) and periodontitis are common chronic inflammatory diseases. However, the association between AD and periodontitis remains poorly understood. This study aimed to evaluate the effects of dental scaling (DS) on the risk of AD among patients with periodontitis.

**Methods:**

In this retrospective cohort study using health insurance data, we identified individuals aged ≥20 years with periodontitis and a matched cohort without a history of periodontitis in Taiwan from 2011 to 2015. Age- and sex-matching was applied to select controls (ratio = 1:1). Both cohorts were followed until the end of 2017 to monitor atopic dermatitis (AD) incidence. Adjusted hazard ratios (HRs) and 95% confidence intervals (CIs) for AD risk associated with periodontitis were estimated using multivariate Cox regression. Among patients with periodontitis, we compared the risk of AD between those who received DS and those who did not.

**Results:**

During the follow-up period, patients with periodontitis had an increased risk of AD compared with those without periodontitis (HR 2.47, 95% CI 2.25–2.71). The association between periodontitis and increased risk of AD was significant in men (HR 2.68, 95% CI 2.33–3.08), women (HR 2.35, 95% CI 2.07–2.66), and people in every age group. Among patients with periodontitis (n = 38,943), DS was associated with a reduced risk of AD (HR 0.33, 95% CI 0.30–0.37), and there was a dose-response relationship (p < 0.0001). The beneficial effects of DS on the risk of AD were observed across subgroups. The risk of AD was lowest in patients with periodontitis who received DS more than four times compared with those without DS (HR 0.14, 95% CI 0.08–0.25).

**Conclusions:**

In conclusion, our study revealed a significant association between periodontitis and increased risk of atopic dermatitis in Taiwanese adults. Moreover, regular dental scaling may lower this risk, underscoring the value of integrating oral care into managing systemic inflammation.

## Introduction

Atopic dermatitis (AD) is a common chronic inflammatory skin disease with major feature of intense pruritus and recurrent eczematous lesions [1]. It was estimated that the prevalence of AD ranged from 1.2 to 17.1% in adults and 0.96 to 22.6% in children all over the world [2]. The etiology and mechanism of AD is complex and multiplex, which caused by interactions of genetic, environmental, and immunological factors [3,4]. Among these, skin microbial abnormalities with *Staphylococcus aureus* colonization (dysbiosis) may play a driving factor in the development of AD [5,6]. Moreover, cutaneous inflammation is also crucial to the pathogenesis of AD, and previous studies reported that AD is strongly associated with other immune-mediated inflammatory diseases, such as alopecia areata, vitiligo, rheumatoid arthritis, systematic lupus erythematosus and inflammatory bowel disease [7,8].

Periodontitis is the chronic immune and inflammatory responses of the supporting tissue of the teeth caused by bacterial infection in the dental biofilm [9,10]. The bibliometric and visual analysis highlighted the detrimental impact of periodontitis on various systemic conditions, particularly nutritional and cardiovascular diseases [11]. Several studies have shown that the connection between chronic periodontitis and the increased risk of systemic diseases, such as cardiovascular disease [12], diabetes mellitus [13], respiratory disease [14], and rheumatoid arthritis [15]. The association between poor oral health status (such as oral symptoms, like sensitive teeth, toothache, bleeding gums or gum pain) and atopic diseases (such as asthma, allergic rhinitis, and AD) was also investigated in Korean adolescents [16–18]. The increased level of pro-inflammatory cytokines were thought to play a crucial role in the pathogenesis [10,19,20]. Notably, non-surgical mechanical debridement and full-mouth disinfection are effective approaches for reducing periodontitis [21].

Periodontitis and AD are both systemic inflammation and immune responses. The dysbiosis in the oral cavity due to periodontitis can lead to elevated levels of pro-inflammatory cytokines, which may contribute to the inflammatory environment observed in AD. Moreover, regular preventive dental treatments, such as dental scaling (DS), have been shown to reduce inflammatory markers and the risk of cardiovascular events, suggesting a potential benefit for other inflammatory conditions like AD [22].

However, limited information was available regarding the association between AD and periodontitis [23,24]. The null hypotheses of this study stated that there was no significant difference in the risk of AD between individuals with and without periodontitis, and that DS had no significant effect on the risk of AD among patients with periodontitis. Utilizing a large-scale health insurance database, this study aimed to investigate the association between periodontitis and the risk of AD. More importantly, we aimed to evaluate the potential preventive effect of DS on the risk of AD among patients with periodontitis.

## Methods

### Source of data

This retrospective cohort study utilized a randomly selected sample of one million individuals enrolled in Taiwan’s Health Insurance program. Comprehensive details regarding the NHI database have been thoroughly documented in our previous publications [25,26]. Established in March 1995, Taiwan’s NHI is a universal, single-payer healthcare system, now covering over 99% of the country’s 23 million residents. The database compiles exhaustive records of inpatient and outpatient healthcare services, including demographic information, primary and secondary diagnostic codes, procedures performed, prescriptions issued, and associated healthcare costs.

This study was conducted using retrospective data obtained from a research database, which our research team accessed only after all personally identifiable information had been fully anonymized and scrambled to protect individual privacy. We accessed the current research database and conducted data analysis on November 25, 2021. In compliance with data protection and privacy regulations, the information on beneficiaries was irreversibly de-identified prior to access, ensuring complete confidentiality. The study protocol was reviewed and approved by the Joint Institutional Review Board of Taipei Medical University (TMU-JIRB-202203134; TMU-JIRB-202006057), in accordance with the ethical principles outlined in the Declaration of Helsinki. The Joint Institutional Review Board of Taipei Medical University also granted a waiver of informed consent, as permitted by the regulations of the Ministry of Health and Welfare in Taiwan, given that no identifiable personal data were involved and the risk to participants was minimal. All research procedures were carried out in strict accordance with applicable institutional guidelines and regulatory requirements.

### Study design

The investigation was structured as a retrospective cohort study, drawing on a random sample of one million insured individuals. From this cohort, we identified 38,934 patients aged over 20 years who were diagnosed with periodontitis between 2011 and 2015. These individuals constituted the exposed cohort. A comparison group of 38,934 individuals without any recorded history of periodontitis was selected using frequency matching by age and sex, ensuring an approximately 1:1 ratio between exposed and unexposed subjects. To guarantee the inclusion of periodontitis cases, we applied a two-year washout period prior to diagnosis, ensuring no prior records of periodontitis during that interval.

To enhance diagnostic accuracy, cases were classified as periodontitis only if dental services were rendered with periodontitis listed as the primary diagnosis. All participants were confirmed to be free of AD at baseline. Follow-up commenced at the index date and continued until either the end of 2017 or censoring due to death. Incident AD, as diagnosed during the follow-up period, served as the primary outcome.

### Definition and criteria

Patients with periodontitis were identified based on dental clinical diagnoses recorded using the International Classification of Diseases, Ninth Revision, Clinical Modification (ICD-9-CM). Additional comorbidities assessed in this study included mental disorders, hypertension, diabetes mellitus, hyperlipidemia, ischemic heart disease, cerebrovascular disease, chronic obstructive pulmonary disease, liver cirrhosis, heart failure, and Parkinson’s disease. Baseline socioeconomic and medical characteristics such as receipt of renal dialysis and low-income status were also considered. These variables were included as covariates or potential confounders in the multivariate regression models, consistent with established literature [1,12–16].

According to Taiwan’s Health Insurance program, DS includes localized and full-mouth scaling for the general population, as well as for individuals with xerostomia, pregnant women, patients with diabetes, those at high risk for dental diseases, and persons with special needs (S1 Table). In clinical settings, DS is routinely performed by dentists using ultrasonic instruments for periodontal maintenance or prophylaxis in Taiwan. Under current policies of Taiwan’s Health Insurance program, individuals aged 13 years and older are eligible for one reimbursed DS session every six months, regardless of whether they have a diagnosis of periodontitis. In other words, DS is available twice a year for all eligible beneficiaries under the health insurance coverage.

The primary exposure under investigation was DS. For the DS cohort, follow-up began on the date of the first DS procedure following the diagnosis of periodontitis. The procedure codes for DS were provided in S1 Table. In contrast, for the non-DS group, follow-up started at the date of diagnosis. This staggered follow-up strategy was employed to prevent immortal time bias, which can lead to an overestimation of treatment effectiveness. Follow-up duration, calculated in person-years, was determined individually for each participant. The incidence of AD was then compared between the DS and non-DS groups. To increase diagnostic specificity, cases of AD were confirmed only when there was at least one outpatient visit in which a dermatologist listed AD as the principal diagnosis.

### Statistical analyses

Baseline characteristics of individuals with and without periodontitis were compared using chi-square tests for categorical variables and t-tests for continuous variables. Similarly, comparisons between periodontitis patients with and without DS were conducted using the same statistical tests. Cox proportional hazards regression models were applied to estimate adjusted hazard ratios (HRs) and corresponding 95% confidence intervals (CIs) for the association between periodontitis and the risk of developing AD. Among individuals with periodontitis, the impact of DS on AD risk was further assessed using adjusted Cox models. Stratified analyses were also conducted to estimate HRs and 95% CIs within subgroups defined by age, sex, and comorbid conditions. All statistical computations were performed using SAS software, version 9.2 (SAS Institute Incorporated, Cary, North Carolina, United States of America).

## Results

In Table 1, there were no significant differences in age or sex between subjects with and without periodontitis because of frequency matching procedure. The proportions of mental disorders, hypertension, diabetes, hyperlipidemia, ischemic heart disease, stroke, chronic obstructive pulmonary disease, liver cirrhosis, heart failure, and DS were higher among people with periodontitis than among people without periodontitis (all p < 0.05).

**Table 1:** Characteristics of people with and without periodontitis.

	No periodontitis (N = 38934)		Periodontitis (N = 38934)		p-value
Sex	n	(%)	n	(%)	1.0000
Female	17367	(44.6)	17367	(44.6)	
Male	21567	(55.4)	21567	(55.4)	
Age, years					1.0000
20-29	7766	(20.0)	7766	(20.0)	
30-39	9356	(24.0)	9356	(24.0)	
40-49	7564	(19.4)	7564	(19.4)	
50-59	6134	(15.8)	6134	(15.8)	
60-69	3970	(10.2)	3970	(10.2)	
70-79	2447	(6.3)	2447	(6.3)	
≥ 80	1697	(4.4)	1697	(4.4)	
Low income					<0.0001
No	37778	(97.0)	38431	(98.7)	
Yes	1156	(3.0)	503	(1.3)	
Coexisting medical conditions					
Mental disorders	7529	(19.3)	8428	(21.7)	<0.0001
Hypertension	6027	(15.5)	6983	(17.9)	<0.0001
Diabetes	3277	(8.4)	3981	(10.2)	<0.0001
Hyperlipidemia	1939	(5.0)	3135	(8.1)	<0.0001
Ischemic heart disease	2122	(5.5)	2907	(7.5)	<0.0001
COPD	1482	(3.8)	1338	(3.4)	0.0057
Liver cirrhosis	771	(2.0)	899	(2.3)	0.0015
Stroke	744	(1.9)	624	(1.6)	0.0011
Heart failure	573	(1.5)	475	(1.2)	0.0023
Parkinson’s disease	326	(0.8)	359	(0.9)	0.2054
Renal dialysis	495	(1.3)	190	(0.5)	<0.0001
Dental scaling					<0.0001
0	26843	(68.9)	4426	(11.4)	
1	7026	(18.1)	5786	(14.9)	
2	2620	(6.7)	5668	(14.6)	
3	1161	(3.0)	5304	(13.6)	
≥ 4	1284	(3.3)	17750	(45.6)	

COPD, chronic obstructive pulmonary disease

In a multivariable Cox proportional hazard model (Table 2), the HR of AD for people with periodontitis were 2.47 (95% CI 2.25–2.71; p < 0.0001) during the follow-up period compared with people had no periodontitis. Periodontitis was a significant factor associated with AD in women (HR 2.35, 95% CI 2.07–2.66; p < 0.0001), men (HR 2.68, 95% CI 2.33–3.08; p < 0.0001), and people aged 20–29 years (HR 2.69, 95% CI 2.23–3.25; p < 0.0001), 30–39 years (HR 2.35, 95% CI 1.96–2.83; p < 0.0001), 40–49 years (HR 2.73, 95% CI 2.16–3.45; p < 0.0001), 50–59 years (HR 2.50, 95% CI 1.90–3.28), 60–69 years (HR 2.77, 95% CI 2.03–3.79; p < 0.0001), and ≥70 years (HR 1.96, 95% CI 1.48–2.59; p < 0.0001). Among people with low income, periodontitis increased the highest risk of AD (HR 3.90, 95% CI 2.18–6.98; p < 0.0001).

**Table 2:** Risk of atopic dermatitis in association with periodontitis in the Cox proportion hazard regression models.

	N	Events	Person-years	Incidence^†^	HR	(95% CI)^*^
No Periodontitis	38934	1069	131666	8.1	1.00	(reference)
Periodontitis	38934	1469	178303	8.2	2.47	(2.25-2.71)
Female						
No Periodontitis	17367	596	58761	10.1	1.00	(reference)
Periodontitis	17367	779	78914	9.9	2.35	(2.07-2.66)
Male						
No Periodontitis	21567	473	72905	6.5	1.00	(reference)
Periodontitis	21567	690	99389	6.9	2.68	(2.33-3.08)
Age, 20–29 years						
No Periodontitis	7766	239	26710	8.9	1.00	(reference)
Periodontitis	7766	333	34584	9.6	2.69	(2.23-3.25)
Age, 30–39 years						
No Periodontitis	9356	267	32591	8.2	1.00	(reference)
Periodontitis	9356	360	42847	8.4	2.35	(1.96-2.83)
Age, 40–49 years						
No Periodontitis	7564	166	25738	6.4	1.00	(reference)
Periodontitis	7564	250	35881	7.0	2.73	(2.16-3.45)
Age, 50–59 years						
No Periodontitis	6134	140	20419	6.9	1.00	(reference)
Periodontitis	6134	181	28973	6.2	2.50	(1.90-3.28)
Age, 60–69 years						
No Periodontitis	3970	102	12490	8.2	1.00	(reference)
Periodontitis	3970	151	17880	8.4	2.77	(2.03-3.79)
Age, ≥ 70 years						
No Periodontitis	4144	155	13718	11.3	1.00	(reference)
Periodontitis	4144	194	18138	10.7	1.96	(1.48-2.59)
No low income						
No Periodontitis	37778	1035	127970	8.1	1.00	(reference)
Periodontitis	38431	1437	176091	8.2	2.44	(2.22-2.68)
Low income						
No Periodontitis	1156	34	3696	9.2	1.00	(reference)
Periodontitis	503	32	2212	14.5	3.90	(2.18-6.98)
0 medical condition						
No Periodontitis	23085	654	72201	9.1	1.00	(reference)
Periodontitis	21327	879	94910	9.3	2.70	(2.40-3.03)
1 medical condition						
No Periodontitis	9853	269	34908	7.7	1.00	(reference)
Periodontitis	10143	356	47067	7.6	2.30	(1.90-2.78)
2 medical conditions						
No Periodontitis	3691	81	14438	5.6	1.00	(reference)
Periodontitis	4540	143	21672	6.6	2.05	(1.44-2.90)
3 medical conditions						
No Periodontitis	1503	41	6351	6.5	1.00	(reference)
Periodontitis	1945	54	9658	5.6	1.14	(0.65-1.99)
≥4 medical conditions						
No Periodontitis	802	24	3768	6.4	1.00	(reference)
Periodontitis	979	37	4996	7.4	1.46	(0.71-3.02)
0 dental scaling						
No Periodontitis	26843	836	83852	10.0	1.00	(reference)
Periodontitis	4426	412	16530	24.9	2.56	(2.27-2.88)
1 dental scaling						
No Periodontitis	7026	149	25258	5.9	1.00	(reference)
Periodontitis	5786	349	22890	15.2	2.67	(2.20-3.25)
2 dental scaling						
No Periodontitis	2620	53	10727	4.9	1.00	(reference)
Periodontitis	5668	231	23493	9.8	2.04	(1.51-2.76)
3 dental scaling						
No Periodontitis	1161	19	5209	3.6	1.00	(reference)
Periodontitis	5304	159	22788	7.0	1.92	(1.18-3.11)
≥4 dental scaling						
No Periodontitis	1284	12	6620	1.8	1.00	(reference)
Periodontitis	17750	318	92602	3.4	1.96	(1.10-3.50)

CI, confidence interval; HR, hazard ratio.

*Adjusted for all covariates listed in Table 1.

†Per 1000 person-years.

The association between periodontitis and AD was significant in people with 0 medical condition (HR 2.70, 95% CI 2.40–3.03; p < 0.0001), 1 medical condition (HR 2.30, 95% CI 1.90–2.78; p < 0.0001), and 2 medical conditions (HR 2.05, 95% CI 1.44–2.90; p < 0.0001). S2 Table showed the stratified analysis of specific medical conditions on the risk of AD associated with periodontitis using the Cox proportional hazard regression models.

Among 38934 patients with periodontitis, 88.6% of them had received DS during the follow-up period (Table 3). Patients with DS had higher proportions of mental disorders (p < 0.0001), hyperlipidemia (p < 0.0001), and ischemic heart disease (p < 0.0001) compared to patients without DS. Patients with DS were younger than patients without DS, and they also had lower proportions of low income (p < 0.0001), stroke (p < 0.0001), heart failure (p < 0.0001), Parkinson’s disease (p = 0.0276), and renal dialysis (p = 0.0090).

**Table 3:** Characteristics of periodontitis patients with and without dental scaling (N = 38934).

	No dental scaling (N = 4426)		Dental scaling (N = 34508)		p-value
Sex	n	(%)	n	(%)	0.0240
Female	1904	(43.0)	15463	(44.8)	
Male	2522	(57.0)	19045	(55.2)	
Age, years					<0.0001
20-29	791	(17.9)	6975	(20.2)	
30-39	1058	(23.9)	8298	(24.1)	
40-49	812	(18.4)	6752	(19.6)	
50-59	628	(14.2)	5506	(16.0)	
60-69	440	(9.9)	3530	(10.2)	
70-79	335	(7.6)	2112	(6.1)	
≥ 80	362	(8.2)	1335	(3.9)	
Low income					<0.0001
No	4338	(98.0)	34093	(98.8)	
Yes	88	(2.0)	415	(1.2)	
Coexisting medical conditions					
Mental disorders	835	(18.9)	7593	(22.0)	<0.0001
Hypertension	833	(18.8)	6150	(17.8)	0.1030
Diabetes	486	(11.0)	3495	(10.1)	0.0780
Hyperlipidemia	263	(5.9)	2872	(8.3)	<0.0001
Ischemic heart disease	289	(6.5)	2618	(7.6)	0.0118
COPD	164	(3.7)	1174	(3.4)	0.2971
Liver cirrhosis	90	(2.0)	809	(2.3)	0.1947
Stroke	107	(2.4)	517	(1.5)	<0.0001
Heart failure	89	(2.0)	386	(1.1)	<0.0001
Parkinson’s disease	54	(1.2)	305	(0.9)	0.0276
Renal dialysis	33	(0.8)	157	(0.5)	0.0090

COPD, chronic obstructive pulmonary disease

After adjusted for the covariates (Table 4), periodontitis patients with DS had reduced risk of AD than patients had no DS (HR 0.33, 95% CI 0.30–0.37; p < 0.0001). The further stratified analysis showed that DS was associated with reduced risk of AD in every subgroup of age, sex, and low income. The association between DS and decreased risk of AD was also significant in patients had no medical condition (HR 0.26, 95% CI 0.22–0.29; p < 0.0001) and in patients had 1 medical condition (HR 0.40, 95% CI 0.31–0.51; p < 0.0001). In Table 5, there was a dose-response relationship between frequency of DS and reduced AD risk (p < 0.0001). The adjusted HRs of 1 visit, 2 visits, 3 visits, and ≥4 visits of DS associated with AD risk were 0.63 (95% CI 0.52–0.77; p < 0.0001), 0.40 (95% CI 0.30–0.54; p < 0.0001), 0.29 (95% CI 0.18–0.47; p < 0.0001), and 0.14 (95% CI 0.08–0.25; p < 0.0001), respectively. S3 Table showed the subgroup analysis for the effects of DS on the risk of AD among patients without periodontitis. The more frequency of DS was associated with reduced risk of AD among patients with no periodontitis (S4 Table). S5 Table showed the immunological profiles of people with and without AD.

**Table 4:** The subgroup analysis for the effects of dental scaling on the risk of atopic dermatitis among patients with periodontitis (N = 38934).

Periodontitis patients		N	Events	Person-years	Incidence^†^	HR	(95% CI)^*^
No DS		4426	412	16530	24.9	1.00	(reference)
DS		34508	1057	129219	8.2	0.33	(0.30-0.37)
Female	No DS	1904	212	6963	30.4	1.00	(reference)
	DS	15463	567	57697	9.8	0.31	(0.27-0.37)
Male	No DS	2522	200	9567	20.9	1.00	(reference)
	DS	19045	490	71522	6.9	0.34	(0.29-0.40)
Age, 20–29 years	No DS	791	95	2858	33.2	1.00	(reference)
	DS	6975	238	25129	9.5	0.26	(0.21-0.34)
Age, 30–39 years	No DS	1058	103	4030	25.6	1.00	(reference)
	DS	8298	257	30558	8.4	0.32	(0.25-0.40)
Age, 40–49 years	No DS	812	75	3138	23.9	1.00	(reference)
	DS	6752	175	26249	6.7	0.27	(0.21-0.36)
Age, 50–59 years	No DS	628	47	2367	19.9	1.00	(reference)
	DS	5506	134	21531	6.2	0.31	(0.22-0.44)
Age, 60–69 years	No DS	440	38	1599	23.8	1.00	(reference)
	DS	3530	113	13119	8.6	0.37	(0.26-0.54)
Age, ≥ 70 years	No DS	697	54	2538	21.3	1.00	(reference)
	DS	3447	140	12633	11.1	0.52	(0.38-0.72)
No low income	No DS	4338	400	16209	24.7	1.00	(reference)
	DS	34093	1037	127735	8.1	0.33	(0.30-0.37)
Low income	No DS	88	12	321	37.4	1.00	(reference)
	DS	415	20	1484	13.5	0.31	(0.15-0.66)
0 medical condition	No DS	2498	294	8949	32.9	1.00	(reference)
	DS	18829	585	68074	8.6	0.26	(0.22-0.29)
1 medical condition	No DS	1083	85	4079	20.8	1.00	(reference)
	DS	9060	271	34437	7.9	0.40	(0.31-0.51)
2 medical conditions	No DS	525	19	2078	9.1	1.00	(reference)
	DS	4015	124	15939	7.8	0.90	(0.55-1.46)
3 medical conditions	No DS	203	6	888	6.8	1.00	(reference)
	DS	1742	48	7118	6.7	1.08	(0.46-2.55)
≥4 medical conditions	No DS	117	8	535	15.0	1.00	(reference)
	DS	862	29	3652	7.9	0.59	(0.27-1.30)

CI, confidence interval; DS, dental scaling; HR, hazard ratio

*Adjusted for all covariates listed in Table 3.

†Per 1000 person-years.

**Table 5:** The effects of dental scaling frequency on the risk of atopic dermatitis among patients with periodontitis (N = 38934).

Frequency of dental scaling	N	Events	Person-years	Incidence^†^	HR	(95% CI)^*^
No dental scaling	4426	412	16530	24.9	1.00	(reference)
1 visit of dental scaling	5786	349	22890	15.2	0.63	(0.52-0.77)
2 visits of dental scaling	5668	231	23493	9.8	0.40	(0.30-0.54)
3 visits of dental scaling	5304	159	22788	7.0	0.29	(0.18-0.47)
≥4 visits of dental scaling	17750	318	92602	3.4	0.14	(0.08-0.25)

CI, confidence interval; HR, hazard ratio.

*Adjusted for all covariates listed in Table 3. P-value <0.0001 for the trend of frequency of dental scaling associated with risk of atopic dermatitis.

†Per 1000 person-years.

## Discussion

The null hypotheses of this study stated that there was no significant difference in the risk of atopic dermatitis between individuals with and without periodontitis, and that dental scaling had no significant effect on the risk of atopic dermatitis among patients with periodontitis. The results showed that patients with periodontitis had a 2.4-fold increased risk of developing AD compared with those without periodontitis. Periodontitis was associated with AD across both sexes, age groups, and individuals with less medical conditions. Our analysis also revealed that patients with periodontitis who received DS experienced a 67% reduction in the risk of AD. The reduced risk was correlated with the frequency of DS, making our study the first to demonstrate this relationship.

Accumulating evidence suggested that oxidative stress played a pivotal role in the pathogenesis of periodontitis by stimulating the production of reactive oxygen species, which contributed to local tissue degradation and initiated systemic inflammatory pathways [27]. Elevated levels of oxidative stress biomarkers were consistently reported in patients with periodontitis and were associated with increased concentrations of pro-inflammatory cytokines, potentially promoting the development of systemic diseases [28]. Concurrently, oxidative stress was also recognized as a key driver in the pathophysiology of AD, where it damaged cutaneous cellular structures, exacerbated inflammation, compromised the skin barrier, and heightened susceptibility to microbial invasion. These pathological processes jointly contributed to both the initiation and progression of AD [29,30].

The potential preventive effect of dental scaling on AD among patients with periodontitis remains incompletely understood. To clarify the findings of this study, at least three possible explanations are provided as follows. First, poor oral conditions can lead to the development of chronic oral infection, causing periodontal diseases. Previous studies reported that periodontal diseases, especially chronic periodontitis, were strongly associated with systemic diseases and several skin disorders [31–33]. One probable mechanism is linked to the systemic inflammation caused by periodontal diseases. Several studies have proposed that chronic inflammatory state of periodontal disease elicited an increase in several inflammation biomarkers, such as C-reactive protein, interleukin-1, interleukin-2, interleukin-6, and tumor necrosis factor-alpha, which could contribute to systemic inflammatory burden [9,10,34]. The Korean study also found that the odds ratios for asthma, allergic rhinitis, and AD, were significantly higher in adolescents with poor oral health due to inflammatory and immunological responses [16]. Moreover, one study revealed that higher levels of inflammatory cytokines such as C-reactive protein, interleukin-6, and tumor necrosis factor-alpha were observed in the elderly population [35]. Therefore, elderly individuals, especially those in the over-70 age group, with higher levels of inflammatory cytokines, contribute less to the inflammatory state caused by periodontitis, and have a lower hazard ratio for atopic dermatitis compared to younger groups. Past studies further demonstrate that in mice, AD exacerbates periodontitis, and the severity of periodontitis increases with the worsening of dermatitis [24,36]. Therefore, the relationship between AD and periodontitis may be reflected in inflammatory responses and cytokine regulation.

Second, other studies indicated that the link between periodontitis and AD was related to the absence of filaggrin [17,37]. Filaggrin is a major structural protein functioning for tight epidermal barrier against allergens and microbiome composition. Mutations of filaggrin gene cause epidermal barrier dysfunction, and has been shown strongly associated with development of AD [3,38]. Meta-analyses of studies reported that the significant odds ratio for AD in filaggrin mutations group compared to control group [39]. There was a cohort study also revealed that the frequency of filaggrin mutations in the development of AD was 31.4%, and none in healthy control in Han Chinese population [40], suggesting filaggrin mutations are important predisposing factors for AD. Such pathogenic mechanism may be occurred in oral cavity, since filaggrin is expressed not only in the skin but also in the human oral mucosa [41]. The barrier defects and increased susceptibility to infections caused by filaggrin mutations could consequently induce periodontal diseases, especially associated with colonization by species of *Aggregatibacter actinomycetemcomitans* and *Porphyromonas gingivalis* [42,43].

Third, skin microbial abnormalities (dysbiosis) is also a hallmark of AD, which not only implicates colonization of *Staphylococcus spp*, but also other microbes such as *Propionibacterium and Malassezia* [5]. Previous studies revealed that the connection between oral cavity and skin microbiota in patients with AD. A whole metagenome profiling demonstrated that the majority of species enriched in AD-susceptible patients are known opportunistic pathogens or commensals of the oral cavity [44]. Meanwhile, the microbiota between the skin and oral cavity of AD patients was found closer and more similar than that that found in healthy individuals [45]. In summary, aforementioned mechanism could be the possible mechanisms for the higher incidence of developing atopic dermatitis in patients with periodontitis.

Under the coverage of Taiwan’s Health Insurance, people can receive dental care service of DS without any copayment in every six months. Interestingly, our study found that the incidence of AD in patients with periodontitis decreased after receiving DS management, which was also observed in several studies showing that oral hygiene care such as frequent tooth brushing, dental prophylaxis including DS, or other periodontitis treatment could reduce the incidence of cardiovascular events including ischemic stroke and myocardial infarction in Taiwanese and Korean populations [22,46]. DS, which could eliminate dental plaque, calculus, and biofilm of both supra- and subgingival deposits, had been proven to lower the incidence of gingivitis, periodontitis and tooth loss [47]. One national population based study in London revealed that lower frequency of tooth brushing was associated with increased level of C reactive protein and fibrinogen [48]. Periodontal treatment not only decreased C-reactive protein concentration but also the level of interleukin-6 and tumor necrosis factor-alpha [49], which was hypothesized to be the mechanism between DS and lower risk of periodontitis. However, the protective effect of DS on the risk of AD may be lower in patients with periodontitis who aged more than 70 years in the current study. A previous study also showed that DS was not associated with decreased risks of acute myocardial infarction or total cardiovascular events in the elderly [22]. The possible explanation is that older patients had more medical conditions and comorbidities, leading to higher levels of inflammatory markers [35], which limited the beneficial effect of DS on the risk of AD.

Our study is characterized by several key strengths. First, we used large population-based insurance data to investigate the effects of DS on the risk of AD among patients with periodontitis. A large sample size can increase statistical power, reduce the margin of error, and improve the precision and generalizability of the results, thereby making the conclusions more robust and less susceptible to random variation. Second, we used Taiwan’s Health Insurance Database, which has covered more than 99% of the population since its initiation 30 years ago. The reliability and validity of this database have been confirmed and widely accepted in numerous scientific studies and journals [22,25,26]. Third, we calculated person-years during the follow-up period only after patients received DS in the DS cohort. This approach helps to avoid immortal time bias, which commonly arises in observational studies involving exposure classification. Furthermore, to the best of our knowledge, this is the first population-based study to report a significant association between DS and a decreased risk of AD in people with periodontitis.

There are some limitations in the present study. First, we used insurance database which lacked information of lifestyle, health behaviours, dietary factors and physical activity. Thus it is difficult to evaluate the oral hygiene care behavior such as frequency of tooth brushing and associated dental symptoms in patients. Second, we also have no information regarding the severity, duration, lesion positions, etiologies, and family history of AD in Taiwan’s Health Insurance Research Database. The number of AD cases may have been underestimated because not all patients with AD seek medical treatment. Thirdly, our findings cannot not be extrapolated to Western populations because there were differences in climate, environment, genetics, dietary, and healthcare between Chinese population and Western population [50–53]. Another limitation of this study is that we could not confirm whether all cases of periodontitis and AD were newly diagnosed by physicians. Since the washout periods for both conditions were limited to two years at baseline, this may have resulted in the inclusion of patients with pre-existing diagnoses. We have acknowledged this as a limitation in this study. Furthermore, In this study, the definition of periodontitis was based on diagnoses made by dentists during dental care visits. This approach may have introduced misclassification bias, as individuals with mild periodontitis who did not seek dental care could have been categorized as non-periodontitis patients. Such misclassification may have led to an underestimation of the true association between periodontitis and the risk of AD, as some individuals with undiagnosed mild periodontitis may have been included in the non-periodontitis group. In addition, the details items of clinical examinations (such as biochemical data and physical measures, risk scores) were not available in this insurance database thus we could not evaluate these influences on our interesting exposure and outcome in this study. Finally, our current research is an observational study that could not provide the solid causal evidence for the associations of periodontitis, DS and risk of AD.

In conclusion, while periodontitis was associated with an elevated risk of AD among Taiwanese adults, people with periodontitis who underwent DS experienced a reduced risk of developing AD. This finding suggests that maintaining regular DS may serve as a modifiable preventive strategy to mitigate the risk of AD in individuals affected by periodontitis. Further research is warranted to explore the underlying mechanisms and to evaluate whether similar protective effects can be observed in other populations.

## Supporting information

S1 Table The definition, codes, and payments points of dental scaling in Taiwan’s Health Insurance Program. (DOC)

S2 Table Stratified analysis of specific medical conditions on the risk of atopic dermatitis associated with periodontitis using the Cox proportional hazard regression models. (DOC)

S3 Table The subgroup analysis for the effects of dental scaling on the risk of atopic dermatitis among patients without periodontitis (N = 38934). (DOC)

S4 Table The effects of dental scaling frequency on the risk of atopic dermatitis among patients with no periodontitis (N = 38934). (DOC)

S5 Table The immunological profiles of people with and without AD. (DOC)

## Abbreviations

AD: atopic dermatitis
CI: conﬁdence interval
HR: hazard ratio.
