# Supplementary material for: Risk of atopic dermatitis in periodontitis patients with and without dental scaling: A retrospective cohort study
Source: PLoS One. 2025 Oct 15;20(10):e0333877. doi: 10.1371/journal.pone.0333877 (PMC12527181; doi:10.1371/journal.pone.0333877)
Supplement: S1 Table — (DOC) [file pone.0333877.s001.doc]

| **Table S1** The definition, codes, and payments points of dental scaling in Taiwan’s Health Insurance Program | | |
| --- | --- | --- |
| Codes | Name of procedure | Payments points |
| 91003C | Scaling: localized | 165 |
| 91004C | Scaling: full mouth | 660 |
| 91005C | Full mouth scaling for xerostomia patients | 720 |
| 91017C | Full mouth scaling for pregnant women | 920 |
| 91089C | Full mouth scaling for patients with diabetes mellitus | 820 |
| 91090C | Full mouth scaling for patients at high risk for dental diseases | 820 |
| 91103C | Scaling for patients with special needs: localized | 165 |
| 91104C | Scaling for patients with special needs: full mouth | 660 |
