# Supplementary material for: Risk of atopic dermatitis in periodontitis patients with and without dental scaling: A retrospective cohort study
Source: PLoS One. 2025 Oct 15;20(10):e0333877. doi: 10.1371/journal.pone.0333877 (PMC12527181; doi:10.1371/journal.pone.0333877)
Supplement: S2 Table — (DOC) [file pone.0333877.s002.doc]

| **Table S2** Stratified analysis of specific medical conditions on the risk of atopic dermatitis associated with periodontitis using the Cox proportional hazard regression models | | | | | | |
| --- | --- | --- | --- | --- | --- | --- |
|  | N | Events | Person-years | Incidence† | HR | (95% CI)* |
| No mental disorders |  |  |  |  |  |  |
| No Periodontitis | 51227 | 2124 | 220391 | 9.6 | 1.00 | (reference) |
| Periodontitis | 53162 | 2122 | 240733 | 8.8 | 1.52 | (1.43-1.62) |
| Mental disorders |  |  |  |  |  |  |
| No Periodontitis | 16334 | 503 | 75310 | 6.7 | 1.00 | (reference) |
| Periodontitis | 14399 | 429 | 69507 | 6.2 | 1.30 | (1.13-1.49) |
| No hypertension |  |  |  |  |  |  |
| No Periodontitis | 55689 | 2205 | 241368 | 9.1 | 1.00 | (reference) |
| Periodontitis | 55978 | 2192 | 253884 | 8.6 | 1.50 | (1.41-1.60) |
| Hypertension |  |  |  |  |  |  |
| No Periodontitis | 11872 | 422 | 54333 | 7.8 | 1.00 | (reference) |
| Periodontitis | 11583 | 359 | 56356 | 6.4 | 1.28 | (1.10-1.50) |
| No diabetes |  |  |  |  |  |  |
| No Periodontitis | 61183 | 2402 | 266739 | 9.0 | 1.00 | (reference) |
| Periodontitis | 60853 | 2368 | 277780 | 8.5 | 1.52 | (1.44-1.62) |
| Diabetes |  |  |  |  |  |  |
| No Periodontitis | 6378 | 225 | 28962 | 7.8 | 1.00 | (reference) |
| Periodontitis | 6708 | 183 | 32460 | 5.6 | 0.98 | (0.79-1.22) |
| No hyperlipidemia |  |  |  |  |  |  |
| No Periodontitis | 62210 | 2460 | 270456 | 9.1 | 1.00 | (reference) |
| Periodontitis | 61736 | 2419 | 281242 | 8.6 | 1.52 | (1.43-1.61) |
| Hyperlipidemia |  |  |  |  |  |  |
| No Periodontitis | 5351 | 167 | 25244 | 6.6 | 1.00 | (reference) |
| Periodontitis | 5825 | 132 | 28998 | 4.6 | 0.91 | (0.71-1.17) |
| No IHD |  |  |  |  |  |  |
| No Periodontitis | 62903 | 2500 | 273749 | 9.1 | 1.00 | (reference) |
| Periodontitis | 62907 | 2425 | 287147 | 8.4 | 1.49 | (1.40-1.58) |
| IHD |  |  |  |  |  |  |
| No Periodontitis | 4658 | 127 | 21952 | 5.8 | 1.00 | (reference) |
| Periodontitis | 4654 | 126 | 23093 | 5.5 | 1.26 | (0.97-1.65) |
| No COPD |  |  |  |  |  |  |
| No Periodontitis | 64330 | 2521 | 280516 | 9.0 | 1.00 | (reference) |
| Periodontitis | 65009 | 2470 | 297378 | 8.3 | 1.49 | (1.40-1.58) |
| COPD |  |  |  |  |  |  |
| No Periodontitis | 3231 | 106 | 15185 | 7.0 | 1.00 | (reference) |
| Periodontitis | 2552 | 81 | 12862 | 6.3 | 1.16 | (0.84-1.60) |
| No liver cirrhosis |  |  |  |  |  |  |
| No Periodontitis | 65692 | 2574 | 286927 | 9.0 | 1.00 | (reference) |
| Periodontitis | 65948 | 2501 | 302369 | 8.3 | 1.48 | (1.40-1.57) |
| Liver cirrhosis |  |  |  |  |  |  |
| No Periodontitis | 1869 | 53 | 8773 | 6.0 | 1.00 | (reference) |
| Periodontitis | 1613 | 50 | 7871 | 6.4 | 1.58 | (1.04-2.40) |
| No stroke |  |  |  |  |  |  |
| No Periodontitis | 66199 | 2595 | 289141 | 9.0 | 1.00 | (reference) |
| Periodontitis | 66504 | 2528 | 304833 | 8.3 | 1.48 | (1.40-1.57) |
| Stroke |  |  |  |  |  |  |
| No Periodontitis | 1362 | 32 | 6559 | 4.9 | 1.00 | (reference) |
| Periodontitis | 1057 | 23 | 5406 | 4.3 | 1.38 | (0.76-2.53) |
| No heart failure |  |  |  |  |  |  |
| No Periodontitis | 66507 | 2599 | 290733 | 8.9 | 1.00 | (reference) |
| Periodontitis | 66869 | 2539 | 306736 | 8.3 | 1.49 | (1.41-1.58) |
| Heart failure |  |  |  |  |  |  |
| No Periodontitis | 1054 | 28 | 4967 | 5.6 | 1.00 | (reference) |
| Periodontitis | 692 | 12 | 3504 | 3.4 | 0.61 | (0.28-1.32) |
| No PD |  |  |  |  |  |  |
| No Periodontitis | 66899 | 2607 | 292646 | 8.9 | 1.00 | (reference) |
| Periodontitis | 67001 | 2540 | 307459 | 8.3 | 1.49 | (1.40-1.57) |
| PD |  |  |  |  |  |  |
| No Periodontitis | 662 | 20 | 3054 | 6.5 | 1.00 | (reference) |
| Periodontitis | 560 | 11 | 2781 | 4.0 | 0.94 | (0.42-2.14) |
| No renal dialysis |  |  |  |  |  |  |
| No Periodontitis | 66867 | 2606 | 292704 | 8.9 | 1.00 | (reference) |
| Periodontitis | 67280 | 2544 | 308912 | 8.2 | 1.48 | (1.40-1.57) |
| Renal dialysis |  |  |  |  |  |  |
| No Periodontitis | 694 | 21 | 2997 | 7.0 | 1.00 | (reference) |
| Periodontitis | 281 | 7 | 1328 | 5.3 | 0.93 | (0.35-2.42) |
| CI, confidence interval; HR, hazard ratio.  *Adjusted for all covariates listed in Table 1.  †Per 1000 person-years. | | | | | | |
