# Supplementary material for: Risk of atopic dermatitis in periodontitis patients with and without dental scaling: A retrospective cohort study
Source: PLoS One. 2025 Oct 15;20(10):e0333877. doi: 10.1371/journal.pone.0333877 (PMC12527181; doi:10.1371/journal.pone.0333877)
Supplement: S3 Table — (DOC) [file pone.0333877.s003.doc]

| **Table S3** The subgroup analysis for the effects of dental scaling on the risk of atopic dermatitis among patients without periodontitis (N=38934) | | | |
| --- | --- | --- | --- |
| Patients without periodontitis | | HR | (95% CI)* |
| No DS | | 1.00 | (reference) |
| DS | | 0.64 | (0.58-0.71) |
| Female | No DS | 1.00 | (reference) |
|  | DS | 0.42 | (0.36-0.48) |
| Male | No DS | 1.00 | (reference) |
|  | DS | 0.53 | (0.45-0.62) |
| Age, 20-29 years | No DS | 1.00 | (reference) |
|  | DS | 0.30 | (0.24-0.37) |
| Age, 30-39 years | No DS | 1.00 | (reference) |
|  | DS | 0.58 | (0.47-0.72) |
| Age, 40-49 years | No DS | 1.00 | (reference) |
|  | DS | 0.53 | (0.41-0.68) |
| Age, 50-59 years | No DS | 1.00 | (reference) |
|  | DS | 0.40 | (0.31-0.54) |
| Age, 60-69 years | No DS | 1.00 | (reference) |
|  | DS | 0.51 | (0.36-0.71) |
| Age, ≥70 years | No DS | 1.00 | (reference) |
|  | DS | 0.68 | (0.49-0.94) |
| No low income | No DS | 1.00 | (reference) |
|  | DS | 0.47 | (0.42-0.52) |
| Low income | No DS | 1.00 | (reference) |
|  | DS | 0.43 | (0.27-0.69) |
| 0 medical condition | No DS | 1.00 | (reference) |
|  | DS | 0.40 | (0.35-0.45) |
| 1 medical condition | No DS | 1.00 | (reference) |
|  | DS | 0.52 | (0.42-0.65) |
| 2 medical conditions | No DS | 1.00 | (reference) |
|  | DS | 0.57 | (0.40-0.81) |
| 3 medical conditions | No DS | 1.00 | (reference) |
|  | DS | 1.09 | (0.66-1.81) |
| ≥ 4medical conditions | No DS | 1.00 | (reference) |
|  | DS | 0.86 | (0.44-1.69) |
| CI, confidence interval; DS, dental scaling; HR, hazard ratio.  *Adjusted for all covariates listed in Table 1. | | | |
