# Supplementary material for: Risk of atopic dermatitis in periodontitis patients with and without dental scaling: A retrospective cohort study
Source: PLoS One. 2025 Oct 15;20(10):e0333877. doi: 10.1371/journal.pone.0333877 (PMC12527181; doi:10.1371/journal.pone.0333877)
Supplement: S4 Table — (DOC) [file pone.0333877.s004.doc]

| **Table S4** The effects of dental scaling frequency on the risk of atopic dermatitis among patients with no periodontitis (N=38934) | | |
| --- | --- | --- |
| Frequency of dental scaling | HR | (95% CI)* |
| No dental scaling | 1.00 | (reference) |
| 1 visit of dental scaling | 1.27 | (1.12-1.45) |
| 2 visits of dental scaling | 0.74 | (0.63-0.86) |
| 3 visits of dental scaling | 0.45 | (0.36-0.56) |
| ≥4 visits of dental scaling | 0.27 | (0.22-0.33) |
| CI, confidence interval; HR, hazard ratio.  *Adjusted for all covariates listed in Table 1. | | |
