# Supplementary material for: Risk of atopic dermatitis in periodontitis patients with and without dental scaling: A retrospective cohort study
Source: PLoS One. 2025 Oct 15;20(10):e0333877. doi: 10.1371/journal.pone.0333877 (PMC12527181; doi:10.1371/journal.pone.0333877)
Supplement: S5 Table — (DOC) [file pone.0333877.s005.doc]

| **Table S5** The immunological profiles of people with and without AD | | | | | |
| --- | --- | --- | --- | --- | --- |
|  | No AD  (N=129944) | | AD  (N=5178) | | p-value |
| Immunological profiles | n | (%) | n | (%) |  |
| Asthma | 4397 | (3.4) | 209 | (4.0) | 0.0112 |
| Rheumatoid arthritis | 879 | (0.7) | 33 | (0.6) | 0.7359 |
| Food allergy | 229 | (0.2) | 5 | (0.1) | 0.1763 |
| AD, atopic dermatitis | | | | | |
